# The role of Internal Solitary Waves on deep-water sedimentary processes: the case of up-slope migrating sediment waves off the Messina Strait

**DOI:** 10.1038/srep36376

**Published:** 2016-11-03

**Authors:** R. Droghei, F. Falcini, D. Casalbore, E. Martorelli, R. Mosetti, G. Sannino, R. Santoleri, F. L. Chiocci

**Affiliations:** 1 CNR-ISAC, Rome, Italy; 2 CNR-IGAG, Rome, Italy; 3 OGS, Trieste, Italy; 4 ENEA, Rome, Italy; 5 University of Rome “La Sapienza”, Rome, Italy

## Abstract

Subaqueous, asymmetric sand waves are typically observed in marine channel/canyon systems, tidal environments, and continental slopes exposed to strong currents, where they are formed by current shear resulting from a dominant unidirectional flow. However, sand-wave fields may be readily observed in marine environments where no such current exists; the physical processes driving their formation are enigmatic or not well understood. We propose that internal solitary waves (ISWs) induced by tides can produce an effective, unidirectional boundary “current” that forms asymmetric sand waves. We test this idea by examining a sand-wave field off the Messina Strait, where we hypothesize that ISWs formed at the interface between intermediate and surface waters are refracted by topography. Hence, we argue that the deflected pattern (i.e., the depth-dependent orientation) of the sand-wave field is due to refraction of such ISWs. Combining field observations and numerical modelling, we show that ISWs can account for three key features: ISWs produce fluid velocities capable of mobilizing bottom sediments; the predicted refraction pattern resulting from the interaction of ISWs with bottom topography matches the observed deflection of the sand waves; and predicted migration rates of sand waves match empirical estimates. This work shows how ISWs may contribute to sculpting the structure of continental margins and it represents a promising link between the geological and oceanographic communities.

Both south and north approaches of the Messina Strait (Mediterranean Sea) are characterized by the presence of sand-wave fields (Fig. 1) that mark the strong tidal processes affecting this region^1^. The Strait connects the Ionian Sea with the Tyrrhenian Sea and is, indeed, an amphidromic point for the semi-diurnal tide of these two sub-basins (Fig. 1). The phase opposition of these tides, along with topographic constraints, forces very intense tidal currents that reach 3 m/s at the sill region^1^. This tidal mechanism causes a periodic (semi-diurnal), northward flux of the denser Levantine Intermediate Water (LIW) underneath the Tyrrhenian surface water (TSW) (Fig. 2; see Supplementary Information).

The sand-wave field we focus on forms off the north entrance of the Messina Strait, between ~200 m and ~300 m depth, running along a topographic mound offshore of Capo Rasocolmo (Fig. 1). This mound is 6–7 km wide, 100–150 m high and extends for about 10 km along a SW-NE direction; seafloor sampling recovered mostly sand (mean grain size ~0.5 mm) along its southern flank^2^. The intriguing feature that makes the sand-wave field worth investigating is the deflected orientation of the bedforms, which run transversally along the 275 m isobath and climbs upwards with a counter-clockwise pattern, until crossing the 200 m isobath (Fig. 1). The sand waves cover an area of about 3 km^2^ and have wavelengths of 60–120 m, wave heights of 1.5–5 m, and lateral extent of 100–300 m; in general, wave dimensions tend to increase northward (Fig. 1).

The sand-wave field was first reported by Selli *et al.* [ref. 3] and Colantoni [ref. 4], and its morphodynamic is relevant for pipelines stability^2^,^5^. Monitoring of a pipeline that brings Algerian gas to the European mainland revealed that some portions of the pipe are exposed above wave troughs while other segments are buried under sandy-gravelly sediment, providing evidence that the sand-waves are mobile^2^,^5^. These studies hypothesize a migration rate from 10^−2^ to 1 m/year, based on semi-quantitative considerations^2^,^5^. Santoro *et al.* [ref. 5] report differences in the geometry of the sand waves among repetitive bathymetric surveys conducted in 1991 and 2001. The height of the bedforms increased approximately twofold everywhere in the field, whereas their wavelength decreased at depth lesser than 218 m, suggesting reworking and/or migration of sand-waves. The sand-wave field is characterized by coarse sediment with grain size that ranges from coarse sand to gravel, suggesting the presence of high bottom velocities able to mobilize the grains and remove finer material^3^,^5^.

Although setting an interesting problem, these works did not attempt to make a mechanistic connection between oceanographic and sedimentary processes in explaining the formation of the sand-wave field. In fact, Selli *et al.* [ref. 3] and Colantoni [ref. 4] suggested that the presence of the sand waves was due to tidal-induced bottom currents. Santoro *et al.* [ref. 5] attributed this bottom current to a combination of tidal, upwelling, and thermohaline effects, and suggested that the migration velocity of the sand waves decreases with increasing depths due to differences in the hydrodynamic forcing. None of these hypotheses, however, takes into consideration the role of internal waves propagating along the LIW-TSW interface^6^,^7^,^8^ and their shoaling effects^9^,^10^.

## Refracting Internal Solitary Waves for sand-waves generation and migration

Sand waves generally form in those tidal environments where the interaction between seafloor morphology and tidal currents is strong enough to allow for the necessary shear stress that triggers sediment motion^11^,^12^,^13^,^14^,^15^. Sand-wave formation can be also due to downslope-flowing turbidity currents, along-slope bottom currents, and internal (progressive or solitary) waves^9^,^10^,^16^,^17^. Although the investigated region is a tidal environment, also characterized by a strong thermohaline circulation^1^, both location and spatial pattern of the sand waves do not suggest the action of either tidal or quasi-geostrophic currents as the main cause of sand motion: i) the sand-wave field is located in a deep region, rather far from the sill; ii) the northward thermohaline flow of the LIW current that would eventually form sand waves does not exceed 20 cm/s [ref. 8] and its known to flow geostrophically on the eastern side of the Tyrrhenian approach of the Strait^18^,^19^; iii) both tidal and/or thermohaline bottom currents cannot justify the deflected pattern of the studied sand waves; iv) the eventual oscillatory velocity field induced by tidal currents and/or internal progressive waves would not explain the asymmetric shape of the sand waves; and iv) there is no reason why the sand-wave size would decrease with decreasing water depth, as observed in the field^3^,^4^.

We argue that the process behind such an intriguing depth-dependent orientation of the asymmetric sand waves must be related to the presence of Internal Solitary Waves (ISWs) that travel northward form the sill of the Messina Strait^6^,^7^,^8^ (Fig. 2; see Supplementary Information). These ISWs (i.e., large-amplitude internal gravity waves generated by the interaction of tidal currents with the sill of the Strait) are triggered when the denser Ionian water suddenly debouches in the less dense Tyrrhenian water: internal gravity waves generate in the Messina Strait by the interaction of barotropic tidal currents with the bathymetry in well-stratified water conditions; under certain circumstances, the internal tide can transform into a set of high frequency, non-linear internal waves (Fig. 2); internal waves dynamics is strongly ruled by the nonlinearity of the phenomenon (which would lead to a breaking wave) and by the dispersion of the media; when these two effects are balanced, coherent structures emerge from an initial disturbance and travel as Internal Solitary Waves (ISWs), also called solitons^7^.

**Figure 1:**
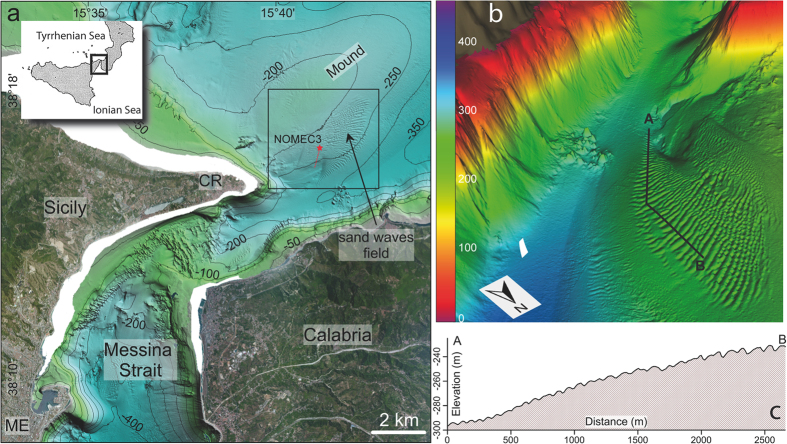
The Messina Strait and the studied sand-wave field. (**a**) Bathymetric map of the Messina Strait (map generated by SURFER 10.2 http://www.goldensoftware.com/products/surfer). The box shows the sand-wave field over the topographic mound off Capo Rasocolmo (CR); the red star indicates position of the NOMEC3 current-meter and the dashed red line indicates orientation of the reference system of the current-meter data (See Fig. 2d). In plan-view, sand-waves have linear or slightly sinuous crest-lines that are mostly oriented obliquely to the isobaths; their crest-lines are laterally continuous southward (close to the Messina Strait), whereas they become more discontinuous northwards. The location of the Messina Strait (central Mediterranean Sea) is shown in the upper-left panel. (**b**) 3D zoom of the sand-wave field (map generated by CARIS Easy View 4.1 http://www.caris.com/products/easy-view). The black line indicates the bathymetric section in (**c)**. (**c**) Bathymetric section of sand-wave crests. Sand waves show variable cross-sections, from asymmetric (longer stoss side followed by shorter and steeper lee side) to more symmetric moving northward.

**Figure 2:**
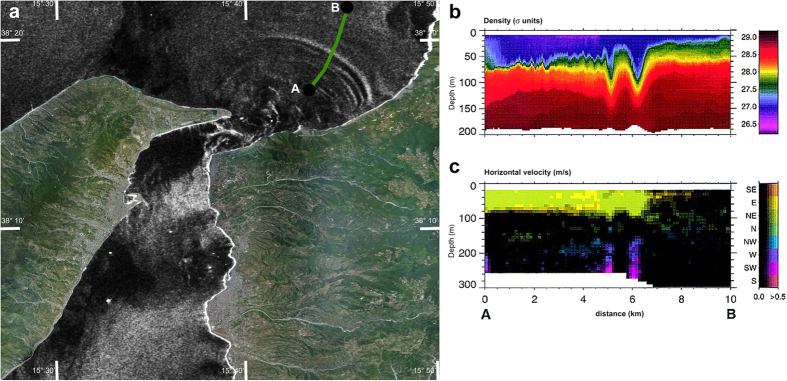
ISW in the Messina Strait. (**a**) COSMO-SkyMed SAR image showing the sea surface manifestations of a train of ISWs propagating northward into the Tyrrhenian Sea (map generated by CARIS Easy View 4.1 http://www.caris.com/products/easy-view). The green line indicates the transect in (**b,c**). (**b**) Density distribution measured by Brandt *et al.* [1999] with a CTD chain between the positions A and B in (**a)**. (**c**) Horizontal velocity field measured by Brandt *et al.* [1999] with the ADCP. Colors represent the different directions of the horizontal velocity, whereas their brightness represents the velocity magnitude. Note the two velocity peaks at the sea bottom right below the ISWs trough. Panels (**b**,**c**) are modified and republished with permission of the American Meteorological Society, from “Evidence for the influence of Atlantic-Ionian stream fluctuations on the tidally induced internal dynamics in the Strait of Messina”, Brandt *et al.*, 29(5), 1999; permission conveyed through Copyright Clearance Center, Inc.

**Figure 3:**
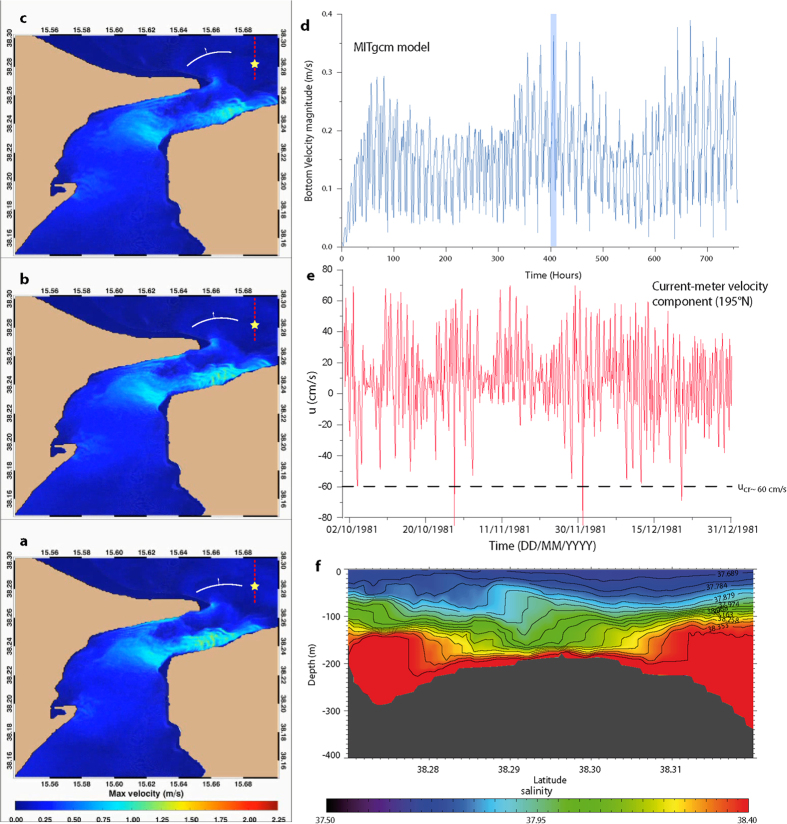
Observations and model results. (**a–c**) Snapshots from the MITgcm numerical simulation showing the bottom velocity magnitude during the northward tidal current (See panel (**d)** and Movie S1 in Supplementary Information). The white arch shows the passage of the refracting ISW over the topographic mound (see Fig. 1a). The yellow star indicates position of the NOMEC3 current-meter. The dashed red line corresponds to the vertical transect in (**f)**. Maps are generated by IDL 8.0 (www.harrisgeospatial.com/IntelliEarthSolutions/GeospatialProducts/ IDL.aspx). (**d**) Bottom velocity field for the MITgcm numerical simulation over the sand waves field. The vertical shaded (blue) bar indicates the passage of the ISW showed in panel (**a–c**) and (**f)**). (**e**) Time series of the velocity magnitude (velocity component toward 195°N) recorded by the NOMEC3 current-meter (from 1 October 1981 to 31 December 1981). The two extremely large peaks (>80 cm/s) demonstrate the intense northward velocity due to the ISW passage (see orientation of the reference system in Fig. 1a). The dashed line indicates the flow speed that marks the critical value for sediment transport. (**f**) Salinity cross-section along transect in (**a–c**) as obtained from the MITgcm numerical simulation. The isohalines show the passage of a concave-up ISW over the topographic mound. Note that the MITgcm model, being hydrostatic, cannot simulate the change of polarity of the ISW due to the topographic effects.

We therefore explore the idea that ISW refraction caused by interaction with the mound offshore of Capo Rasocolmo (Fig. 1; Supplementary Fig. S1), and thus magnitude and orientation of fluid stresses that would result from passage of ISWs, is at the base of the sand waves formation. The hypothesis of ISWs that can remobilize the bottom sediment according to the spatial pattern of the sand-wave field is supported by current-meter and hydrographic measurements, as well as by a numerical experiment^5^,^8^,^20^ (Fig. 3; Supplementary Movie S1). The two main processes that would explain both the presence and the pattern of the sand-wave field are therefore as follows: i) the sedimentary mound (Fig. 1) would induce wave refraction, deflecting the ISW vector toward shallow areas (Figs 1 and 3, and Supplementary Fig. S1); the trough of the ISW would mainly contribute to the intense (i.e., > 50 cm/s) horizontal velocity at the bottom, triggering sediment motion along the direction of the wave vector^8^ (Figs 2 and 3d, and Supplementary Fig. S2). This latter feature points out the main difference between internal progressive waves and ISWs in moving sand along the wave vector: internal progressive waves would cause an oscillating motion, leading to symmetric sand waves, while the passage of an ISW induces a unidirectional momentum to a fluid parcel^21^,^22^ (Figs 2 and 3, and Supplementary Fig. S2), which agrees with the asymmetric pattern of the sand waves off Capo Rasocolmo.

A theoretical framework for ISWs is the well-known Korteweg-de Vries (KdV) equation, which describes the waveform for weakly dispersive non-linear internal waves propagating in straits of uniform and shallow depth in two-layer fluid^7^,^23^ (Supplementary Information). This model, however, did not consider any uneven topography and the consequent refracting effects of the ISWs. Further generalizations of the KdV model allow for a description of ISWs over a slowly varying topography^24^,^25^,^26^,^27^. In particular, Grimshaw *et al.* [refs 24 and 25] model ISWs behavior near the coast by using the Korteweg- de Vries equations with variable coefficients (i.e., the vKdV and the Gardner e-vKdV equations), which describe the shoaling and deformation of ISWs over the continental shelf. We here use a ray method that allows us to take into account the second horizontal dimension and thus to investigate wave refraction while, along a ray, the generalized KdV equation remains valid for a slowly varying depth^28^.

**Figure 4:**
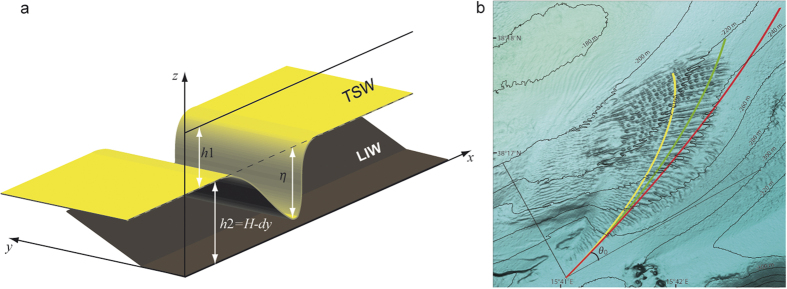
The refracting ISW model. (**a)** 3D sketch of the KdV model and the simulated topography used for equation (3). The yellow surface represents an ISW of amplitude *η*, while *h*_1_ and *h*_2_ are the upper (TSW) and bottom (LIW) layer thicknesses, respectively. Note that *h*_2_ varies along the y-axis due to sea floor topography (brown surface). The dashed line indicates the unperturbed interface between the two layers. (**b**) Results of equation (3) for different initial angle between the ISW vector and the bathymetry in the deepest part of the sand-wave field (map generated by SURFER 10.2 http://www.goldensoftware.com/products/surfer). Yellow curve: *h*_1_ = 149 m, *H* = 151 m, *d* = 8 × 10^−2^; Green curve: *h*_1_ = 149 m, *H* = 151 m, *d* = 5 × 10^−2^; Red curve: *h*_1_ = 100 m, *H* = 200 m, *d* = 5 × 10^−2^. All curves are obtained with *θ*_0_ = Π/2.5 and *g’* = 8 × 10^−2^ m s^−2^. The three curves show that the ISW refraction increases by increasing the topographic slope (*d*) and/or by decreasing the initial bottom layer thickness *H* (See Supplementary Information for a full description of Equation 3 solution). The yellow curve orthogonally runs best along the sand waves since *h*_1_, *H*, and *d* values are very close to the real ones.

In a shallow water approximation, the analytical solution of the KdV equation for a single ISW is (Fig. 3e and Fig. 4a).


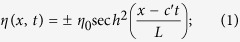


where *c*′ is the non-linear phase speed, *L* is the measure of the width of the soliton solution (i.e., the scale length), and *η*_0_ the initial ISW amplitude (Supplementary Information). We point out here that the phase speed of an ISW depends on the depth ratio between the two layers and the reduce gravity (Fig. 4a), stressing the role of topographic effects on ISW dynamics: a change of bottom layer thickness strongly affects both phase speed and wave polarity (Supplementary Information).

In accordance with the KdV model, particle trajectories induced by the presence of a soliton are not closed during the passage of an ISW, and thus there is no backward motion^21^,^22^ (Figs 2 and 3d, and Supplementary Fig. S2). All particles, therefore, move in the same direction (not exceeding the travelling-wave speed) and the horizontal water velocity in the lower layer is in the opposite direction of that in the upper layer (Supplementary Information). However, according to the vKdV and the Gardner e-vKdV equations on the ISW deformation over a varying topography^25^, a concave-up ISW is affected by shoaling and changes its polarity^24^. Indeed, when ISWs propagate over a variable seafloor, the ensuing topographic gradients along with the current-wave interaction can cause a drastic change in the direction, length, polarity, and height of waves. This phenomenon can be ascribed to the wave refraction over the sloping seafloor^29^. Similar to the refraction occurring on continental shelves^29^,^30^,^31^ the hypothesized process strongly orients the internal-wave crest lines along isobaths (Figs 3a–c and 4b; see also Fig. S1).

## Results

This ISW refraction is described by using a non-linear ray method that leads to a ray equation of linear long wave theory of the type of Snell’s law^28^,^31^


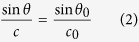


where *θ* is the angle between wave crest and depth contour at an arbitrary depth, *θ*_*0*_ is the angle between wave crest and depth contour in deep-water (Fig. 4), *c* is the wave celerity at an arbitrary depth, and *c*_0_ is the deep-water wave celerity. By defining the ray trajectory as 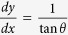 one obtains


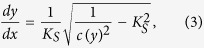


where 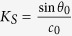 and 
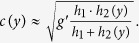


Equation (3) describes the deflection of the ISW vector in terms of *K*_*s*_ and the bottom-dependent wave celerity *c(y*), which in turn depends on the slowly varying bottom layer thickness *h*_2_ = *h*_2_(*y*) (Fig. 3a). The solution of equation (3) gives the trajectory *x* = *x(y*) of the refracted ISW that runs along the sedimentary mound (Fig. 4b) as a function of the initial angle between the ISW vector and the bathymetry in the deepest part of the sand-wave field (Supplementary Information). Numerical, theoretical and current-meter data^8^,^20^,^28^ support our initial hypothesis that the topographically-induced ISW refraction generates the deflected sand-waves pattern (Fig. 3).

Regarding the ISW ability to remobilize sediment and form sand waves, the KdV model demonstrates the presence of bottom velocity peaks underneath the ISW trough 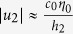
~60 cm/s [ref. 23] over the region where the sand-wave field forms (i.e., from *h*_2_ = 200–100 m). This theoretical estimate is in agreement with *in situ* current-meter data (Fig. 3d and Supplementary Fig. S2) and gives a shear velocity (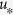 ~ 0.1 m/s) that exceeds the critical shear velocity (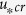 ~ 0.07 m/s) associated to the grain size measured on the sand-wave field^3^ (Supplementary Information). Moreover, these quantities set the proportionality between the height/depth ratio of sand waves dimensionless excess tractive force^15^. By considering a wave height of 5 m it results a water depth of ~100 m, which approximately correspond to the depth of the bottom layer (*h*_2_) (Fig. 2b). We finally stress that bottom velocity usually consists of several components, e.g., barotropic tidal flows, baroclinic internal tides and internal solitary waves. However, current meter data show that the bottom shear velocity due to solitary waves is the dominant component, contributing up to 70% of the whole signal (Fig. 2c and Supplementary Fig. S2).

For large *h*_2_≫*h*_1_, the ISW-induced velocity field would have the opposite direction with respect to the northward wave propagation. However, in shallow conditions (*h*_2_~*h*_1_ or *h*_2_ < *h*_1_) and under the presence of external shear flows (as for the case of the sand-wave field region) the ISW changes its polarity^23^,^32^,^33^,^34^,^35^. The topographic mound off Capo Rasocolmo is therefore expected to affect the concavity of the ISW formed at the sill of the Messina Strait. This is confirmed by current-meter and morphologic data (Figs 1 and 3d) that show a northward (and stronger) sediment flux (i.e., northward bottom velocity peaks and a lee side of sand waves that faces towards north), again in agreement with the theoretical expectations^24^,^25^,^27^.

Knowledge of bottom velocity, critical shear stress, and geometrical characteristics of the sand waves allows for the estimation of migration rate 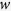, which is given by the relation for sediment flux (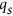) associated with sand waves formation and migration^36^:


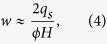


where *ϕ* is a porosity coefficient (~1), *H* is the sediment wave height, and *q*_*s*_ is the sediment flux as obtained by using the revised Mayer-Peter Müller formula^37^ for all bottom velocity events (i.e., those associated to the ISW passage) for which u_*_ – u_*cr_ > 1. From equation (4) and the estimation of 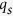 we therefore obtain a migration rate of ~10^−1 ^m/year (Supplementary Information), a value that lays in between the values hypothesized and semi-quantitatively found by previous work^2^,^5^.

## Conclusions

Our results give fundamental explanations regarding: i) driving forces, ii) migration rate, and iii) spatial distribution (i.e., deflected pattern) of the sand waves off Capo Rasocolmo, therefore providing an extremely useful case study for similar sedimentary structures^9^,^10^,^17^. Only the passage of ISWs is able to justify the deflected pattern caused by the refraction process and the unidirectional velocity field associated to the wave trough that explains, in turn, sediment transport and migration rate of the asymmetric sand waves. We therefore demonstrate that this kind of sand waves can be formed by ISWs, so providing a case study to recognize and quantify hydrodynamic drivers for a specific seafloor morphology. This may help to estimate flow conditions from seafloor mapping, an information of large environmental and engineering relevance^38^,^39^.

There are very few studies that put emphasis on the relation between ISWs and sand dunes or, in general, sediment resuspension^17^,^40^. Our findings represent a quantitative study on the role of ISWs for coarse-grained sediment transport in the deep sea that may lead to a better understanding of sedimentary processes and seafloor evolution, which is critical for geohazard assessment^5^,^38^,^39^,^40^,^41^. Although the northern entrance of the Messina Strait is morphologically peculiar, we believe that ISW-induced sand waves would occur worldwide and that they have not yet interpreted in this way. The distinctive formation mechanism related to ISWs was proposed, only recently, for very large sand dunes located on the continental slope of the South China Sea^17^. This therefore reinforce the idea that the occurrence of ISWs-sand waves might have been underestimated.

### Methodology

Multibeam bathymetry in the study area was collected during 5 oceanographic cruises carried out between 2005 and 2009 on board of R/V *Universitatis*-(CoNISMa), R/V *Urania* (CNR), and a small launch in shallow water areas (<−100 m). Data were acquired with multibeam systems working at different frequency (50, 100 and 455 kHz) in order to obtain the full resolution for each bathymetric range. Data were DGPS-positioned for deep water surveys, whereas Real-Time Kinematic positioning was supplied only for shallow water survey. Daily acquisition of sound velocity profiles throughout the water column at 0.1 m depth intervals and patch test surveys for multibeam calibration were realized. Moreover, real-time sound velocity close to the transducer was used as well as tide corrections provided by the nearby Messina tide-gauge station (www.mareografico.it). Raw data were processed using Caris Hips and Sips 6.1 software, encompassing data re-calibration and depth control, application of correct tide, statistical filters and manual editing of each single swath to remove organized/non-organized noise. Data were merged and gridded to obtain Digital Terrain Models (DTMs) with cell size variable from 1 m in shelf sectors (<−100 m) to 10 m in deeper water settings.

The Massachusetts Institute of Technology general circulation model (MITgcm) has been used for this work (See Supplementary Information) in order to test the presence of a refracting solitary wave on the studied region. The model domain covers the Messina Strait from 37°54′ N to 38° 24′ N by means of a non-uniform curvilinear orthogonal grid composed of 300 × 840 points. The spatial resolution of the model grid ranges between 15 and 150 m, with the minimum values reached in the narrowest part of the strait. To adequately resolve the pycnocline, the model uses 55 vertical levels with a thickness of 7.5 m in the upper 300 m, gradually increasing in the remaining 15 levels up to 100 m. The maximum depth reached in the model is 1320 m. Model bathymetry was obtained from a bilinear interpolation of the very-high-resolution multibeam bathymetric data (See Supplementary Information).

Detailed and long (from November 1980 to April 1982) time series of bottom currents in the area of interest have been obtained during a survey conducted by OGS committed by SNAM S.p.A.

## Additional Information

**How to cite this article**: Droghei, R. *et al.* The role of Internal Solitary Waves on deep-water sedimentary processes: the case of up-slope migrating sediment waves off the Messina Strait. *Sci. Rep.* **6**, 36376; doi: 10.1038/srep36376 (2016).

**Publisher’s note**: Springer Nature remains neutral with regard to jurisdictional claims in published maps and institutional affiliations.

## Supplementary Material

Supplementary Information

Supplementary Movie S1
