# Supplementary material for: The role of Internal Solitary Waves on deep-water sedimentary processes: the case of up-slope migrating sediment waves off the Messina Strait
Source: Sci Rep. 2016 Nov 3;6:36376. doi: 10.1038/srep36376 (PMC5093411; doi:10.1038/srep36376)
Supplement: Supplementary Information [file srep36376-s1.pdf]

Supplementary

The role of Internal Solitary Waves on deep-water sedimentary processes: the case of up-slope migrating sediment waves off the Messina Strait

R. Droghei[1], F. Falcini[1], D. Casalbore[2-5], E. Martorelli[2], R. Mosetti[3], G. Sannino[4], R. Santoleri[1], F.L. Chiocci[5].

[1]*CNR-ISAC, Rome, Italy*

[2]*CNR-IGAG, Rome, Italy*

[3]*OGS, Trieste, Italy*

[4]*ENEA, Rome, Italy*

[5]*University of Rome "La Sapienza", Rome, Italy*

Abstract. This document contains supplementary materials including oceanographic and geological settings as well as methodologies and supporting analysis. Section 1 provides additional background information on the Messina Strait hydrographic and bathymetric characteristics, explaining the role of tides and the formation of Internal Solitary Waves (ISWs). Section 2 provides details regarding the sand waves we analyze, including the description of the bathymetric mound where they form. Section 3 contains the derivation of both Korteweg de Vries (KdV) and refracting wave models, describing the role of ISW in forming the sand waves, Finally, Section 4 provides the methodology we use for the determination of sediment transport and sand waves migration rate from in situ current-meter observations.

1. Hydrodynamic settings of the Messina Strait (currents and ISWs)

The Messina Strait separates Sicily from the Italian peninsula (Figure 1). It therefore connects the Tyrrhenian Sea with the Ionian Sea and it is characterized by strong hydrodynamic conditions. The Messina sill is a semidiurnal tidal amphidromic point for the tides of the Tyrrhenian and Ionian Sea tides, which act in opposition to each other. This results in large magnitude velocities that reach up to 3 m/s at the sill region[1-3], due also to the particular bathymetric constraint. Such a pattern leads to a periodic (semi-diurnal) flux of the denser Levantine Intermediate Water (LIW) flowing northward underneath the surface Tyrrhenian water. This bottom current (locally called "rema montante") alternates the so called "rema scendente", i.e., a surface southward flux of Tyrrhenian water in the

opposite direction. Moreover, the wind produces a strong drift current on the surface with upwelling of Ionian water or Tyrrhenian water[4,5]. Once in the Tyrrhenian basin, the tidal branch of LIW flows geostrophically northward along the Calabrian coast at ~ 200-300 m depth[6,7].

Another important phenomenon occurring in the Messina Strait is the presence of internal waves (IWs), that is, gravity waves that oscillate along the interface between two fluid layers of different densities and generate by the interaction of barotropic tidal currents with the bathymetry in well-stratified water bodies (Figure 2). Under certain circumstances, the internal tide can transform into a set of high frequency, non-linear internal waves. IWs dynamics is strongly ruled by the nonlinearity of the phenomenon (which would lead to a breaking wave) and by the dispersion of the media. When these two effects are balanced, coherent structures emerge from an initial disturbance and travel as Internal Solitary Waves (ISWs), also called solitons[8].

ISWs have been observed in coastal ocean through in situ measurements (CDT, XBT, current-meters) and remote sensing (SAR and Ocean Color images; Figure S1). Their importance has increased in the last decade since they seem to be responsible for a significant fraction of the mixing that should maintain the observed ocean circulation[9,10]. Displacement of the thermocline by ISWs can also affect the refraction of sound and has importance for acoustic studies of relevance to the design of sonar systems and acoustic underwater communications. The strong current and shear stress associated with solitons, combined with variable buoyancy effects across interfaces, may also destabilize underwater platform and drilling operations for oil explorations[11]. Furthermore, they provides a mechanism for the removal of energy from the barotropic tide, the internal tide, into the high frequency internal waves, eventually to be dissipated into turbulent motions when the wave break[12].

The evolution of ISW generated by an external forcing like a tide is a topic that attracted much interest in the past[13]. In particular, the downstream evolution of the interface between the two layers, its interaction with the bathymetry, and the effects of it on the velocity field of both layers are the main characteristics that we need to address for our study. A detail analysis of those is provided by the Korteweg de Vries model[14] and by the Snell's law, which are both discussed in Section 3 of this Supplementary Information.

2. *Numerical modeling*

The MITgcm is used for simulating and test the presence of ISWs over the studied region (Supplementary Movie). This model solves the fully nonlinear, non-hydrostatic Navier–Stokes equations under the Boussinesq approximation for an incompressible fluid with a spatial finite-volume discretization on a curvilinear computational grid. The model formulation, which includes implicit free surface and partial step topography, is described in detail by Marshall et al. [ref. 15,16] and its source code and documentation are available at the MITgcm web site (http://mitgcm.org/sealion/online_documents/node2.html). The non-uniform curvilinear orthogonal grid is composed by 300 × 840 points over the Messina Strait domain (from 37°54′ N to 38° 24′ N), with spatial resolution ranging between 15 and 150 m. The model uses 55 vertical levels that increase their thickness with depth (7.5 m in the upper 300 m and 100 m in the remaining 15 levels). The maximum depth reached in the model is 1320 m. Model bathymetry was obtained from a bilinear interpolation of the very-high-resolution multibeam bathymetric data used in Antonioli et al. [ref. 17]. The very high horizontal resolution, together with the partial cell formulation, result in a very detailed description of the bathymetry. No-slip conditions were imposed at the bottom and lateral solid boundaries. The selected tracer advection scheme is a third-order direct space–time flux-limited scheme[18], which is unconditionally stable and does not require additional diffusion. As turbulent closure parametrization for vertical viscosity and diffusivity, we have used the scheme proposed by Pacanowski & Philander [ref. 19]. We emphasize that a similar physical configuration for the MITgcm has been successfully used to study the dynamics of the Strait of Gibraltar[20-22]. The model simulation started from rest, forced at the north and south open boundaries through the specification of the present-day barotropic tidal currents, taking into account the principal semidiurnal and diurnal harmonics (M2, S2, O1 and K1). The amplitude and phase have been computed using the OTIS package[23]. Initial conditions have been extracted from the Medar-MedAtlas climatological Database[24]. A technique similar to that used by Sannino et al. [ref. 25] has been applied to spin up the model: in order to achieve a steady circulation the model is first run without tidal forcing, then it is laterally forced by tidal components to reach a stable time periodic solution. After the spin-up phase, the model is run for a further tropical month (27.321 days) to produce our reference experiment. The same model configuration has been successfully applied recently in a multidisciplinary research aimed to study the timing of emergence of the land bridge between Sicily and mainland Italy during the last 40 kyr [ref. 17].

3. *The Korteweg de Vries and the refracting wave models*

ISWs are commonly generated by a barotropic tidal flow over a varying topography (e.g., a sill) that gives rise to the internal tide. Under certain circumstances, the internal tide can transform into a set of high frequency, non-linear internal waves. A theoretical framework for the interpretation of ISWs was made by Osborne and Burch [ref. 26] and after by Artale et al. [ref. 8], where they make use of well-known KdV equation that describes the waveform for weakly dispersive non-linear internal waves, propagating in straits of uniform and shallow depth in two-layer fluid. However, those works did not consider any uneven topography and the consequent refracting effects of the ISWs. In a two-layer fluid, the dimensional form of the KdV equation is given by

$$\frac{\partial\eta}{\partial t}+c_0\frac{\partial\eta}{\partial x}+\alpha\eta\frac{\partial\eta}{\partial x}+\gamma\frac{\partial^3\eta}{\partial x^3}=0; \qquad \text{(S1)}$$

where $\eta(x,t)$ is the interface displacement between the two fluids (Figure 4a). The coefficients in the equation (S1) are:

$$c_0\cong\sqrt{g'\frac{h_1h_2}{h_1+h_2}}, \qquad \alpha\cong\frac{3}{2}\frac{h_1h_2}{h_1-h_2}c_0, \qquad \gamma\cong\frac{c_0h_1h_2}{6}; \qquad \text{(S2)}$$

where h_j and ρ_j are, respectively, the depth and density of the upper (j=1) and lower (j=2) layers, $g'=g\frac{\Delta\rho}{\rho}$ is the reduced gravity with $\rho \sim \rho_1 \sim \rho_2$ and $\Delta\rho = \rho_1 - \rho_2$. We recall that if $h_1 < h_2$ (i.e., a thin upper layer), then $\alpha < 0$, which results in a downward displacements of the ISW, that is $\eta < 0$..

In a shallow water approximation, and assuming that the upper layer is thinner than the lower one, the analytical solution of the KdV equation for a single ISW is[26]

$$\eta(x,t)=-\eta_0\,\mathrm{sec}\,h^2\left(\frac{x-c't}{L}\right); \qquad \text{(S3)}$$

where c' is the non-linear phase speed and L is the measure of the width of the soliton solution (i.e., the scale length):

$$c'=c_0\left(1-\frac{\eta_0\alpha}{3c_0}\right), \qquad L=\sqrt{-\frac{12\gamma}{\eta_0\alpha}}.$$

Following the KdV model, the horizontal velocities of the water particles of the solitons in the upper and lower layer are given respectively by[26]

$$u_1(x,t) = \frac{c_0\eta_0}{h_1}\operatorname{sec}h^2\left(\frac{x - c't}{L}\right), \qquad u_2(x,t) = -\frac{c_0\eta_0}{h_2}\operatorname{sec}h^2\left(\frac{x - c't}{L}\right), \tag{S4}$$

indicating that velocities in the lower layer are opposite in direction to those in the upper layer.

As regards the refracting effect[27], by setting $\frac{\sin\phi}{c} = \frac{\sin\phi_0}{c_0} = K_S$, where ϕ is the angle between wave crest and depth contour at an arbitrary depth, ϕ_0 is the angle between wave crest and depth contour in deep-water, c is the wave celerity at an arbitrary depth, and c_0 is the deep-water wave celerity, as defined in equation (S2). Defining the ray trajectory as $\frac{dy}{dx} = \frac{1}{\tan\phi}$ one obtains

$$\frac{dy}{dx} = \frac{1}{K_S}\sqrt{\frac{1}{c^2} - K_S^2}\,, \tag{S5}$$

which describes the deflection of the ISW vector in terms of K_S and wave celerity. By assuming a slowly varying topography along the y-direction (Figure 4a) in order to model the effect of the sedimentary mound on the ISW, the depth of the lower layer is a function of the y variable, viz., $h_2 = h_2(y)$. The resulting phase speed of the refracted wave is

$$c(y) \approx \sqrt{g'\frac{h_1 \cdot h_2(y)}{h_1 + h_2(y)}}\,, \tag{S6}$$

which allows to explicitly write equation (S5) as a function of the two layer water depths:

$$\frac{dy}{dx} = \frac{1}{K_S}\sqrt{\frac{h_1 + h_2(y)}{g' h_1 \cdot h_2(y)} - K_S^2}\,. \tag{S7}$$

Hence, we can solve the ODE (S7) by separation of variables method: in the simple case of $h_2(y) = H - d{\cdot}y$ (Figure 3a) the trajectory of the wave direction is given by

$$dx = \frac{dy}{\frac{1}{K_S}\sqrt{\frac{(h_1 + H - d \cdot y)}{g' h_1 \cdot (H - d \cdot y)} - K_S^2}}\,. \tag{S8}$$

The integration of equation (S8) gives the trajectory of the refracted ISW running along the sedimentary mound (Figure 4b). It results that the velocity field u_2 in (S4) is oriented along the

trajectory defined by (S8). This, consequently, explains the formation of the deflected sand-wave field.

4. *Estimating sand waves migration rate*

To estimate the transport capacity of velocity field induced by the passage of the ISW over the sand-wave field we convert the bottom layer velocity (u_2) to shear velocity (u_*) using the law-of-the-wall[28]. Data consist of detailed and long (from November 1980 to April 1982) time series of bottom currents in the area of interest, obtained during a survey conducted by OGS committed by SNAM S.p.A[29]. From a harmonic tidal analysis, the following amplitude have been obtained for the principal constituents: M2=17.6 cm/s; S2= 7.5 cm/s; N2= 3.7 cm/s; Nu2= 1.9; K2 = 1.4 cm/s. K1=5.0 cm/s; O1=2.3 cm/s; P1=2.2 cm/s. The resulting maximum tidal amplitude is of the order of 40 cm/s (Figure S2). Velocity magnitudes from these data agree with the estimated values from the theoretical model (S4), thus confirming the presence of ISW-induced velocity peaks: the residual component (i.e., the non-tidal component that also includes the velocity filed induced by the ISW passage) can contribute from 50 to 70% of the whole signal.

We then recognize all data points for which shear velocity exceeded the critical value for entrainment for the median grain size, D = 0.5 mm and compute sediment transport capacity (i.e., sediment flux) for which ($u_* - u_{*cr}$) > 0 by using the sediment flux obtained from the revised Mayer-Peter Müller formula[30]:

$$\tilde{q}_s = 4.93(\tilde{\tau}_b - 0.0470)^{1.60} \qquad \text{(S9)}$$

Here $\tilde{q}_s = \dfrac{q_s}{D\sqrt{RgD}}$ is non-dimensional, instantaneous sediment volumetric flux per unit width (q_s), where $R = 1.68$ is the submerged specific gravity, and $\tilde{\tau}_b = \dfrac{u_*^{\ 2}}{RgD}$ is the non-dimensional bed shear stress. Consistent with our approach, the value u_* = 0.1 m/s (with u_{*cr} = 0.07 m/s) for the sand waves-forming velocity field is the appropriate choice[31,32] and the resulting instantaneous sediment flux is q_s = 0.2 10^{-5} m^2/s. It is worth noting that ratio u_* / u_{*cr} suggests a bed load transport regime and that these values agrees with the stability field for sand waves (see Figure 10 in Rubin and McCulloch [ref. 33])

To move forward we apply the concept of the “geomorphic work”[34] to the velocity record: the combination of frequency of occurrence and magnitude of transport makes it the dominant velocity bottom layer field in terms of time-averaged sediment transport. This brings to the assumption of a constant “formative” shear stress and (ii) an intermittency factor (I) describing the fraction of time that the formative stress is active[28]. For our sand-wave field, the formative bottom layer velocity occurs approximately 3% of the time, i.e., I = 0.03 [ref. 28]. Consequently, the instantaneous transport equation (S9) can be converted into an annual sand flux $< q_s >= I\, q_s$. Using this procedure, we computed an annual sand flux capacity of $< q_s >= 0.6$ m^2/ yr.

In order to estimate migration rate of the sand waves we use conservation of mass applied to a migrating dune[35]. By assuming that sand waves maintain a constant shape while migrating, and that all the sand eroded from the upwind (stoss) side is deposited on the downwind (lee) side, for a triangular sand-wave shape the sand flux (q_s) can be related to migration rate and sand-wave height by conservation of mass[36]:

$$< q_s > \approx \phi w \frac{H}{2} \qquad \text{(S10)}$$

where ϕ is an order-one correction for sediment porosity, which we assume to be 1 for simplicity, w is the annual migration rate and H the sand-wave height. From the estimated annual sand flux $\langle q_s \rangle$ and by inverting equation (S10) we obtained the annually-averaged migration rate $w \sim 10^{-1}$ m/yr, a value that is in between those hypothesized by Santoro et al.’s works [ref. 37,38].

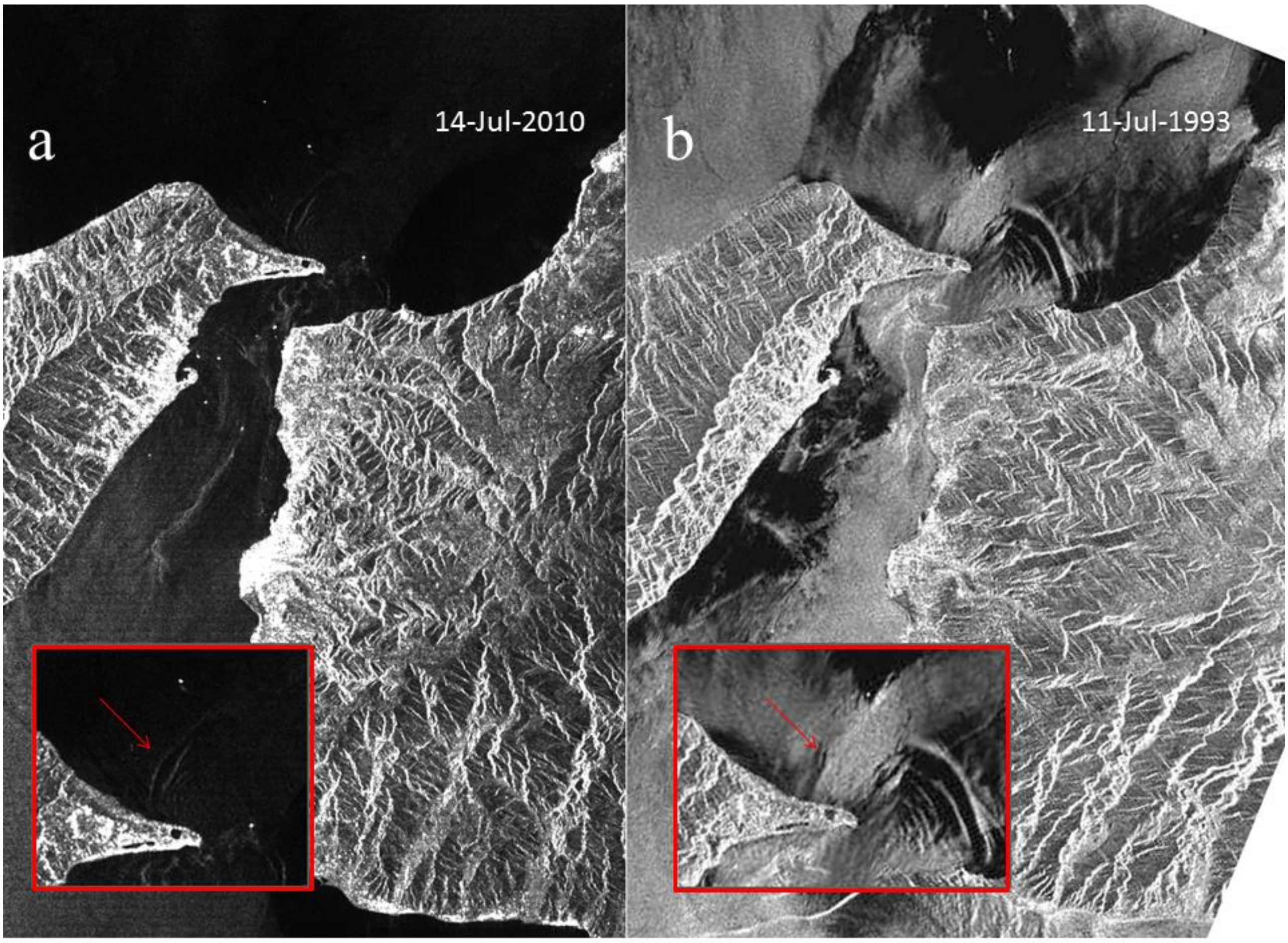

Figure S1 – Refracting ISW in the Messina Strait. SAR images showing the sea surface manifestations of a train of ISWs propagating northward into the Tyrrhenian Sea and, in particular, the refracting "branches" off Capo Rasocolmo (indicated by arrows in the red boxes). **a,** map generated by CARIS Easy View 4.1 http://www.caris.com/products/easy-view. **b,** map downloaded from ESA-Earth Online website https://earth.esa.int/.

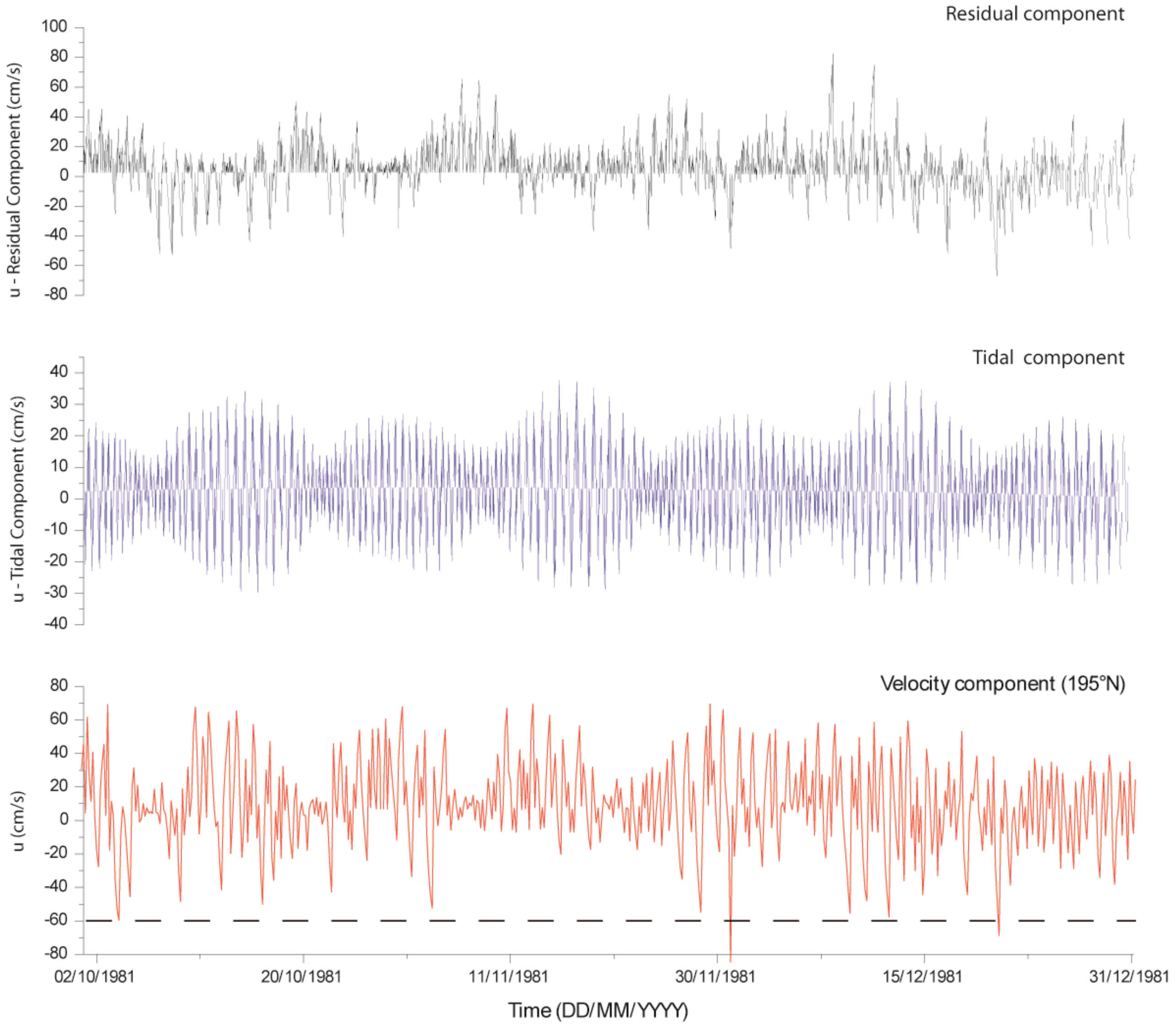

Figure S2 – Current-meter data on the topographic mound off Capo Rasocolmo. Time series of the velocity magnitude (velocity component toward 195°N) recorded by the NOMEC3 current-meter (from 1 October 1981 to 31 December 1981). Red: the whole signal as in Figure 3; Blue: tidal component; Black: residual component, where the strong, northward peaks mark the passage on concave-down ISWs.

Movie S1 – Tidal currents in the Messina Strait. Sea bottom velocity field simulated by the MITgcm numerical model for an entire tidal cycle (map generated by IDL 8.0 http://www.harrisgeospatial.com/IntelliEarthSolutions/GeospatialProducts/IDL.aspx). The presence of ISWs over the Capo Rasocolmo topographic mound is enlightened in Figure 3.

REFERENCES

1. Vercelli, F. (1925). Il regime dello correnti e delle maree nello stretto di Messina. Commissione Internazionale del Mediterraneo, negli Anni 1922 e 1923, 209 pp.

2. Sapia, A., & Salusti, E. (1987). Observation of nonlinear internal solitary wave trains at the northern and southern mouths of the Strait of Messina. Deep Sea Research Part A. Oceanographic Research Papers, 34(7), 1081-1092.

3. Brandt, P., Rubino, A., Quadfasel, D., Alpers, W., Sellschopp, J., & Fiekas, H. V. (1999). Evidence for the influence of Atlantic-Ionian stream fluctuations on the tidally induced internal dynamics in the Strait of Messina. J. Phys. Oceanogr., 29(5), 1071-1080.

4. Defant, A., Scilla e Cariddi e le correnti di marea nello Stretto di Messina, Geof. Pura Appl., 2, 93–112, 1940.

5. Hopkins, T. S., Salusti, E., & Settimi, D. (1984). Tidal forcing of the water mass interface in the Strait of Messina. J. Geophys. Res. Oceans, 89(C2), 2013-2024.

6. Marullo, S., and R. Santoleri. "Fronts and internal currents at the northern mouth of the Strait of Messina." Il Nuovo Cimento C 9, no. 3 (1986): 701-713.

7. Martorelli, E., Falcini, F., Salusti, E., & Chiocci, F. L. (2010). Analysis and modeling of contourite drifts and contour currents off promontories in the Italian Seas (Mediterranean Sea). Marine Geology, 278(1), 19-30.

8. Artale, V., Levi, D., Marullo, S., & Santoleri, R. (1990). Analysis of nonlinear internal waves observed by Landsat thematic mapper. J. Geophys. Res. Oceans (1978–2012), 95(C9), 16065-16073.

9. Garrett, C., & Kunze, E. (2007). Internal tide generation in the deep ocean. *Annu. Rev. Fluid Mech.*, 39, 57-87.

10. Alford, M. H., MacKinnon, J. A., Simmons, H. L., & Nash, J. D. (2016). Near-inertial internal gravity waves in the ocean. *Annual review of marine science,* 8, 95-123.

11. Fraser, N. (1999), Surfing an oil rig, Energy Rev. Feb/Mar, 20– 24.

12. Zhao, Z., Alford, M. H., & Girton, J. B. (2012). Mapping low-mode internal tides from multisatellite altimetry. Oceanography.

13. Maxworthy, T. (1979). A note on the internal solitary waves produced by tidal flow over a three-dimensional ridge. J. Geophys. Res. Oceans (1978–2012), 84(C1), 338-346.

14. Gardner, C. S., Greene, J. M., Kruskal, M. D., & Miura, R. M. (1967). Method for solving the Korteweg-deVries equation. Physical Review Letters, 19(19), 1095

15. Marshall, J., Adcroft, A., Hill, C., Perelman, L. & Heisey, C. (1997a). A finite-volume, incompressible Navier Stokes model for studies of the ocean on parallel computers. J. Geophys. Res., 102, 5753–5766.

16. Marshall, J., Hill, C., Perelman, L. & Adcroft, A. (1997b). Hydrostatic, quasi-hydrostatic, and nonhydrostatic ocean modeling. J. Geophys. Res., 102, 5733–5752.

17. Antonioli F., V. Lo Presti, M. Gasparo Morticelli, L. Bonfiglio, M. Mannino, M. R. Palombo, G. Sannino, L. Ferranti, S. Furlani, K. Lambeck, S. Canese, R. Catalano, F. Latino Chiocci, G. Mangano, G. Scicchitano, R. Tonielli. Latest Pleistocene (40-17 ka) connection between Italy and Sicily: implication for the spread of modern humans. (2015) Geology and Archaeology: Submerged Landscapes of the Continental Shelf - The Geological Society - Special Publications. doi:10.1144/SP411.1. ISBN: 978-1-86239-691-3.

18. Hundsdorfer, W., B. Koren, M. van Loon, and J. G. Verwer (1995), A positive finite-difference advection scheme, J. Comput. Phys., 117, 35–46.

19. Pacanowski, R. C., and S. G. H. Philander (1981), Parameterisation of vertical mixing in numerical models of tropical oceans, J. Phys. Oceanogr., 11, 1443–1451.

20. Sanchez-Garrido, J. C., G. Sannino, L. Liberti, J. Garcia Lafuente, and L. J. Pratt (2011): Numerical modelling of three-dimensional stratified tidal flow over Camarinal Sill, Strait of Gibraltar. J. Geophys. Res., VOL. 116, C12026, doi:10.1029/2011JC007093. ISSN: 21699291.

21. García Lafuente, J., Bruque Pozas, E., Sánchez Garrido, J.C., Sannino, G., Sammartino, S. The interface mixing layer and the tidal dynamics at the eastern part of the Strait of Gibraltar (2013) Journal of Marine Systems, 117-118, pp. 31-42. ISSN: 09247963.

22. Sannino, G., Garrido, J.C.S., Liberti, L., Pratt, L. Exchange flow through the strait of gibraltar as simulated by a s-coordinate hydrostatic model and a z-coordinate non hydrostatic model (2014). In book: The Mediterranean Sea: Temporal Variability and Spatial Patterns. Editors: G. Borzelli, M. Gacic, P. Lionello, P. Malanotte-Rizzoli. American Geophysical Union. pp. 25-50. ISBN: 978-1-118-84734-3.

23. Egbert, G. D., & Erofeeva, S. Y. (2002). Efficient inverse modeling of barotropic ocean tides. Journal of Atmospheric and Oceanic Technology, 19(2), 183-204.

24. Medatlas (2002): Mediterranean and Black Sea Database of Temperature, Salinity and Bio-Chemical Parameters Climatological Atlas, Eur. Comm. Mar. Sci. and Technol. Programme, Paris.

25. Sannino G., A. Carillo, L. Pratt (2009), Hydraulic criticality of the exchange flow through the Strait of Gibraltar, J. Phys. Oceanogr. Vol 39, 11, 2779-2799. DOI:10.1175/2009JPO4075.1. ISSN: 00223670.

26. Osborne, A. R., & Burch, T. L. (1980). Internal solitons in the Andaman Sea. Science, 208(4443), 451-460.

27. Zeng and Alpers, (2004). Generation of internal solitary waves in the Sulu Sea and their refraction by bottom topography studied by ERS SAR imagery and a numerical model. INT. J. REMOTE SENSING, 10–20 APRIL, 2004, VOL. 25, NO. 7–8, 1277–128

28. Jerolmack, D. J., Ewing, R. C., Falcini, F., Martin, R. L., Masteller, C., Phillips, C., ... & Buynevich, I. (2012). Internal boundary layer model for the evolution of desert dune fields. *Nature Geoscience*, 5(3), 206-209.

29. Michelato, A. and Mosetti, R. (1982). "Studio delle correnti di fondo della zona a nord dello Stretto di Messina". Progetto NOMEC (costumer: SNAM S.p.A.).

30. Wong, M., & Parker, G. Reanalysis and correction of bed-load relation of Meyer-Peter and Müller using their own database. *J. Hydraul. Eng.* 132, 1159-1168 (2006).

31. Drake, D. E., Cacchione, D. A., & Grant, W. D. (1992). Shear stress and bed roughness estimates for combined wave and current flows over a rippled bed. *J. Geophys. Res.* Oceans (1978–2012), 97(C2), 2319-2326.

32. Flemming, B. W. (2000, March). The role of grain size, water depth and flow velocity as scaling factors controlling the size of subaqueous dunes. In Marine Sandwave Dynamics, International Workshop (pp. 23-24).

33. Rubin, D.M., and McCulloch, D.S., 1980, Single and superimposed bedforms: A synthesis of San Francisco Bay and flume observations: Sedimentary Geology, v. 26, p. 207-231.

34. Wolman, M. G., & Miller, J. P. (1960). Magnitude and frequency of forces in geomorphic processes. J. Geol. 54-74.

35. Mohrig, D., & Smith, J. D. (1996). Predicting the migration rates of subaqueous dunes. Water Resources Research, 32(10), 3207-3217.

36. Bagnold, R. A. The Physics of Blown Sand and Desert Dunes (Methuen, 1941).

37. Santoro, V. C., Amore, E., Cavallaro, L., Cozzo, G., & Foti, E. (2002). Sand waves in the Messina strait, Italy. J. Coastal Res, 36, 640-653.

38. Santoro, V. C., Amore, E., Cavallaro, L., & De Lauro, M. (2004). Evolution of sand waves in the Messina Strait, Italy. Ocean Dynamics, 54(3-4), 392-398.
